# External validation of pooled cohort equations using systolic blood pressure intervention trial data

**DOI:** 10.1186/s13104-019-4293-1

**Published:** 2019-05-14

**Authors:** Takashi Kuragaichi, Yuki Kataoka, Chisato Miyakoshi, Tadashi Miyamoto, Yukihito Sato

**Affiliations:** 1 Department of Cardiology, Hyogo Prefectural Amagasaki General Medical Center, 2-17-77, Higashinaniwa-Cho, Amagasaki, Hyogo 660-8550 Japan; 2 Hospital Care Research Unit, Hyogo Prefectural Amagasaki General Medical Center, Amagasaki, Hyogo Japan; 3 Department of Pediatrics, Kobe City Medical Center General Hospital, Kobe, Hyogo Japan

**Keywords:** Pooled cohort equations, Calibration, Discrimination, Atherosclerosis, Statin therapy

## Abstract

**Objective:**

The risk of atherosclerotic cardiovascular disease (ASCVD) is estimated using the American College of Cardiology (ACC)/American Heart Association (AHA) Pooled Cohort Equations (PCEs). However, the accuracy of this tool remains controversial, particularly among patients who are recommended statin therapy according to the ACC/AHA guidelines. We performed external validation of PCEs among patients eligible for statin therapy using data from the systolic blood pressure intervention trial (SPRINT).

**Results:**

Our study included 4057 patients from among the 9361 patients in SPRINT. The mean patient age was 64.5 years, and the median predicted 10-year risks of ASCVD were 17.2% and 12.3% for men and women, respectively. Over a median follow-up of 3.3 years, 133 primary events (including 23 cardiovascular deaths) were noted, whereas 304 events were predicted by the PCEs. The PCEs demonstrated poor calibration (Hosmer–Lemeshow test, p < 0.001) and overestimated the probability consistently. Additionally, they showed moderate discrimination [area under the curve: 0.65 (95% confidence interval, 0.60–0.69)]. This study demonstrates that PCEs might overestimate the risk of ASCVD in patients who are recommended statin therapy.

## Introduction

To estimate atherosclerotic cardiovascular disease (ASCVD) risk, the 2013 American College of Cardiology (ACC)/American Heart Association (AHA) guidelines [1] recommended the use of pooled cohort equations (PCEs) derived from four major population-based cohort studies in the United States in the 1990s [2]. However, these equations overestimated the ASCVD risk in three major United States cohorts [3]. Two external validation cohorts, multi ethnic study and atherosclerosis (MESA) [4] and reasons for geographic and racial differences in stroke (REGARDS) [5], observed a similar risk overestimation.

The use of statin therapy for patients whose 10-year ASCVD risk exceeded 5% or 7.5% is recommended by 2013ACC/AHA guidelines [1]. However, this approach has proved controversial, particularly for primary prevention in adults whose low-density lipoprotein cholesterol (LDL-C) level is between 70 and 189 mg/dL but who are not diagnosed with ASCVD or diabetes. The guidelines recommend these patients receive statin treatment; however, it has been reported that their predicted ASCVD risk by the PCEs was systematically higher than the observed risk in the Bioimage study [6], although not in the REGARDS study [5].

The aim of the present study was to conduct an external validation of PCEs among patients eligible for statin therapy using data from the systolic blood pressure intervention trial (SPRINT) [7].

## Main text

### Methods

We conducted a secondary analysis of SPRINT data to evaluate the calibration and discriminatory ability of PCEs. SPRINT was a randomized, controlled, open-label trial sponsored by the National Heart, Lung, and Blood Institute (NHLBI) in a formal data repository that was intended to facilitate data sharing from clinical trials and observational studies (https://biolincc.nhlbi.nih.gov/home/). Patients were enrolled into SPRINT from November 2010 to March 2013 at 102 clinical sites in the United States, including Puerto Rico. The trial was approved by institutional review boards at participating study sites; all patients provided written informed consent [7]. In the analysis of SPRINT data, we received ethical approval from the institutional review board of the Hyogo Prefectural Amagasaki General Medical Center (No. 28-100).

#### Study population

The SPRINT trial included patients who met the following inclusion criteria: age at least 50 years; a systolic blood pressure of 130–180 mmHg; no history of diabetes or stroke; and at least one cardiovascular risk factor (clinical or subclinical cardiovascular disease except stroke or chronic kidney disease), or a 10-year risk of cardiovascular disease ≥ 15% based on the Framingham risk score, or age ≥ 75 years [7]. From these patients, we selected patients who had been recommended to begin taking statins according to the ACC/AHA guidelines [1]. These met the following criteria: age, 40–79 years; no clinical ASCVD events; no statin use at baseline; and LDL-C in the range 70–190 mg/dL (or, if the LDL-C level was unavailable, a non-high-density lipoprotein cholesterol level in the range 100–219 mg/dL). We used the primary SPRINT outcomes (cardiovascular death, myocardial infarction, and stroke) but we excluded heart failure. We applied the PCEs to calculate the predicted 10-year ASCVD risk for each patient. Because the SPRINT trial had not yet completed 10 years of follow-up, we calculated the observed and predicted ASCVD incidences at 5 years. We performed calculations with up to 5 years of follow-up using a baseline hazard adjusted from a 10-year to a 5-year rate, as previously described. [5].

#### Statistical analysis

The calibration of the PCEs was determined using the Hosmer–Lemeshow test and a calibration plot. The discriminatory ability was calculated according to the area under the receiver operating characteristic curve. The 95% confidence interval was calculated by using the bootstrap method. All statistical analyses were performed by using Stata^®^ ver. 13.0 (Stata Corp, College Station, TX, USA) and R 3.3.2 (R foundation for Statistical Computing). Two-sided p-values of < 0.05 were considered statistically significant. Our data analysis used complete-case analysis.

### Results

**Fig. 1:**
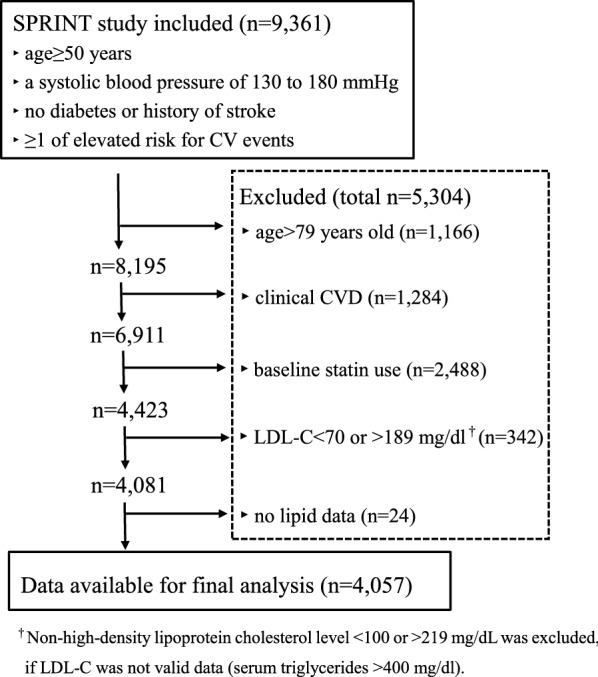
Flowchart of patients who met inclusion criteria for the study

Among the 9361 SPRINT patients, 4057 were included in the final analysis (Fig. 1). Of these, 2467 (60.8%) were males and 1603 (39.5%) were African-Americans. The median predicted 10-year ASCVD risks were 17.2% in men and 12.3% in women. Patients were categorized into four groups based on their 10-year predicted ASCVD risk: < 5%, 5% to less than 7.5%, 7.5% to less than 10%, and ≥ 10% (Table 1). A total of 3126 (77%) patients had a predicted ASCVD risk > 10%. Patients with higher 10-year predicted ASCVD risk were older, and this group included higher percentages of African Americans and men.

**Table 1:** Baseline characteristics in predicted 10-y ASCVD risk (n = 4057)

	< 5%	5–7.5%	7.5–10%	> 10%
Patients no. (%)	165 (4.0)	319 (7.9)	447 (11.0)	3126 (77.0)
Age, mean (SD)	55.1 (3.7)	57.6 (4.4)	59.6 (5.1)	66.4 (7.6)
African-Americans, no. (%)	43 (26.0)	113 (35.4)	198 (44.3)	1249 (40.0)
Men, no. (%)	26 (15.8)	103 (32.3)	199 (44.5)	2139 (68.4)
Systolic blood pressure, mean (SD) (mmHg)	130.0 (15.1)	134.1 (14.0)	134.7 (13.7)	142.8 (15.5)
Anti-hypertensive medication, no. (%)	146 (88.5)	262 (82.1)	370 (82.8)	2726 (87.2)
TC, mean (SD) (mg/dL)	207.5 (33.7)	203.5 (32.9)	200.0 (31.5)	201.1 (31.4)
HDL-C, mean (SD) (mg/dL)	57.4 (18.1)	55.4 (14.9)	54.1 (15.5)	53.0 (15.0)
LDL-C, mean (SD) (mg/dL)	125.2 (26.9)	124.3 (27.6)	122.2 (26.7)	122.9 (27.2)

*TC* total cholesterol, *HDL*-*C* high-density lipoprotein cholesterol, *LDL*-*C* low-density lipoprotein cholesterol

**Fig. 2:**
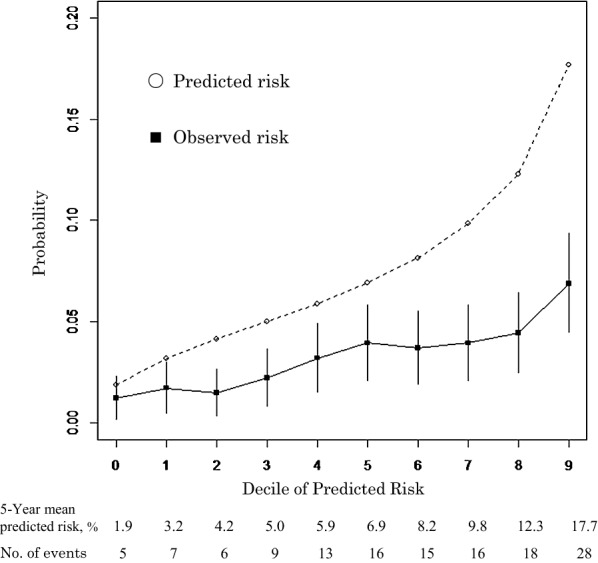
Comparison of observed versus predicted risk

There were 133 primary events (including 23 cardiovascular deaths) observed over a median follow-up of 3.3 years, whereas 304 events were predicted by the PCEs. The PCEs overestimated the risk of cardiovascular events by 128.6%. The calibration plots demonstrated consistent overestimation of the predicted probabilities (Hosmer–Lemeshow test, p < 0.001) according to linear correlation analysis (Fig. 2). The PCEs were only moderately discriminatory toward the ASCVD prediction, with an area under the curve of 0.65 (95% confidence interval: 0.60–0.69).

### Discussion

Our study showed that the PCEs had moderate discriminatory ability, poor calibration, and consistently overestimated the ASCVD risks among patients for whom the ACC/AHA guidelines recommended statin initiation in the SPRINT population. Furthermore, this difference was greater in the patient group with higher predicted ASCVD risk because the observed risk did not necessarily match the predicted risk. In most external validation cohorts in the United States, the PCEs appeared to overestimate the observed risk [8]. However, among the patients considered for statin initiation, whether PCEs were appropriately calibrated for ASCVD events or not is controversial. Indeed, the PCEs showed good calibration in an analysis of the REGARDS study [5] but showed poor calibration in the Bioimage study [6]. Rana et al. [9] reported the PCEs substantially overestimated ASCVD risk in a large multi-ethnic “real world” population. Similarly, our results demonstrated that the PCEs overestimated ASCVD risk in the SPRINT population. This indicates that PCEs overestimates regardless choice of the study population; risk overestimation may be explained by changes in ASCVD rates over time. PCEs derived from the 1990s population data do not reflect the lower current ASCVD rates. Other reasons suggested for the overestimation include more contemporary use of statin therapy, increasing use of revascularization therapies, and under ascertainment of outcomes in selected cohorts [8, 10, 11]. In fact, it was reported the measuring accurate outcomes by comprehensive surveillance reduced overestimation in a Multiethnic Cohort from the Women’s Health Initiative [12]. Consequently, many patients would be inappropriately prescribed statin therapy because of overestimation. Recently, Yadlowsky et al. [13] showed the ‘revised PCEs’ reduced overestimation of need for statins among contemporary populations. Accurate estimation of risk is essential to effectively balance the therapeutic risk and benefit of statins.

### Conclusion

The PCEs overestimated ASCVD risk in patients for whom statin therapy is recommended in the SPRINT population. Risk prediction equations may need to be updated according to changing patterns of risk in contemporary society.

## Limitation

A limitation of this study was the shorter follow-up period, because the SPRINT study was stopped early despite the planned follow-up of 5 years. Although, the SPRINT was a blood pressure-lowering study for high CVD-risk patients, PCEs was calculated the future ASCVD risk using only baseline data, and blood pressure change over time is not considered. Further, since SPRINT study has not been examined for the use of statins or antiplatelet therapy during follow-up, cardiovascular risk may have been modified by the provision of risk-reducing therapies.

## Data Availability

The data that support the findings of this study are available from NHLBI repository (https://biolincc.nhlbi.nih.gov/studies/sprint_pop/), but restrictions apply to the availability of these data, which were used under license for the current study, and so are not publicly available.

## Abbreviations

ACC: American College of Cardiology
AHA: American Heart Association
PCEs: pooled cohort equations
SPRINT: systolic blood pressure intervention trial
ASCVD: atherosclerotic cardiovascular disease
LDL-C: low-density lipoprotein cholesterol
